# 1.5 T MR-guided and daily adapted SBRT for prostate cancer: feasibility, preliminary clinical tolerability, quality of life and patient-reported outcomes during treatment

**DOI:** 10.1186/s13014-020-01510-w

**Published:** 2020-03-23

**Authors:** Filippo Alongi, Michele Rigo, Vanessa Figlia, Francesco Cuccia, Niccolò Giaj-Levra, Luca Nicosia, Francesco Ricchetti, Gianluisa Sicignano, Antonio De Simone, Stefania Naccarato, Ruggero Ruggieri, Rosario Mazzola

**Affiliations:** 1 Advanced Radiation Oncology Department, IRCCS Sacro Cuore Don Calabria Hospital, Cancer Care Center, Don Calabria, Negrar di Valpolicella, 37024 Verona, Italy; 2 University of Brescia, Brescia, Italy

**Keywords:** Prostate cancer, SBRT, MRI-guided radiotherapy, Adaptive radiotherapy, QoL

## Abstract

****Background**:**

Unity Elekta is a unique magnetic resonance (MR)-linac that conjugates a 1.5 Tesla MR unit with a 7 MV flattening filter free accelerator.A prospective observational study for the clinical use of Elekta Unity is currently ongoing in our department. Herein, we present our preliminary report on the feasibility, quality of life, and patient-reported outcomes measures (PROMs) for localized prostate cancer (PC) treated with stereotactic body radiotherapy (SBRT).

****Methods**:**

The SBRT protocol consisted of a 35 Gy schedule delivered in 5 fractions within 2 weeks. Toxicity and quality of life (QoL) were assessed at baseline and after treatment using the Common Terminology Criteria for Adverse Events v5.0, International Prostatic Symptoms Score (IPSS), ICIQ-SF, IIEF-5, and EORTC-QLQ-C30 and PR-25 questionnaires.

****Results**:**

Between October 2019 and January 2020, 25 patients with localized PC were recruited. The median age was 68 years (range, 54–82); 4 were low risk, 11 favorable intermediate risk (IR) and 10 unfavorable IR. Median iPSA was 6.8 ng/ml (range, 1–19), and 9 of these patients (36%) received concurrent androgen deprivation therapy. Median prostate volume was 36 cc (range, 20–61); median baseline IPSS was 5 (range, 0–10). Median time for fraction was 53 min (range, 34–86); adaptive strategy with daily critical structure and target re-contouring and daily replanning (adapt to shape) was performed in all cases. No grade ≥ 3 adverse event was observed, three patients (12%) reported grade 2 acute genitourinary toxicity (urinary frequency, urinary tract pain and urinary retention), while only one patient reported mild rectal pain. No relevant deteriorations were reported in PROMs.

****Conclusion**:**

To the best of our knowledge, this is the first experience reporting feasibility, clinician-reported outcome measurements, and PROMs for 1.5 T MR-guided adaptive SBRT for localized prostate cancer. The preliminary data collected here report optimal safety and excellent tolerability, as also confirmed by PROMs questionnaires. Moreover, the data on technical feasibility and timing of online daily adapted planning and delivery are promising. More mature data are warranted.

**Trial registration:**

Date of approval April 2019 and numbered MRI/LINAC n°23,748.

## Introduction

In the case of localized prostate cancer (PC), active surveillance, surgery and radiotherapy represent viable therapeutic options due to similar long-term oncologic outcomes [1]. According to the treatment received, genitourinary side effects are more pronounced after surgery, whereas rectal toxicity represents the most common adverse event after radical radiotherapy (RT). Finally, the active monitoring can be affected by a higher incidence of distant metastases compared to active therapies, although lower rates of patient-reported harms are described [2]. Furthermore, the effects on quality of life among prostatectomy, 3-dimensional conformal RT and active monitoring were reported by the Protect Trial [1]. 3-dimensional conformal RT has resulted in a limited negative impact on patient reported outcomes (PROMs) of urinary continence, whereas late bowel function has been worse compared to the other Protect Trial arms [3].

Clinical experiences in PC RT by means of intensity-modulated and image-guided techniques (IMRT-IGRT) have demonstrated a lower probability of RT-related adverse events [4–6]. These RT-technological and technical advances have allowed clinicians to deliver higher radiation doses to the target with similarly limited toxicities. To date, moderate PC hypofractionation represents the standard of care [7, 8]. Moreover, since 2014, the National Comprehensive Cancer Network (NCCN) Guidelines [9] have stated that, in centers with appropriate technology and expertise, extreme hypofractionated treatment could be considered as a potential option to offer for selected localized PC. Since then, more robust evidence and new technological platforms are consolidating the role of extreme hypofractionation (also known as stereotactic body radiotherapy - SBRT) for localized PC [10–12].

Elekta (Stockolm, Sweden) Unity is a unique magnetic resonance (MR)-linac that conjugates a 1.5 Tesla MR unit with a 7 MV flattening filter free accelerator mounted on a rotating gantry system, enabling the daily verification of real-time patient anatomy, and allowing daily treatment planning.

A prospective observational study for the clinical use of Elekta Unity is currently ongoing in our department. Herein, we present our preliminary report on the feasibility, quality of life and patient-reported outcomes measures (PROMs) for localized PC treated with stereotactic body radiotherapy (SBRT).

## Materials and methods

The present study depicts the PC subgroup of the ongoing prospective observational study, which had received approval from the local Ethical Committee on April 2019 (MRI/LINAC n°23,748).

The inclusion criteria of the present study were: age > 18 years, Karnofsky index > 70% (Eastern Cooperative Oncology Group (ECOG) Performance Status ≤2), PSA < 20 ng/ml, histologically proven prostate adenocarcinoma, cT1-T2 stage, no pathological lymph nodes on computed tomography (CT) and magnetic resonance imaging (MRI), no distant metastases, no previous prostate surgery other than transurethral resection of the prostate (at least a 6-week interval before the initiation of RT), no malignant tumors in the last 5 years, International Prostate Symptom Score (IPSS) 0–15, combined androgen deprivation therapy (ADT) according to risk category.

The exclusion criteria were: prostate size greater than 80 cc, clinically positive nodes or a lymph node involvement risk > 15%, previous transurethral resection of the prostate (TURP) less than 6 months before RT, previous prostate surgery other than TURP, previous pelvic irradiation, MRI contraindications (electronic devices such as pacemakers, defibrillators, deep brain stimulators, cochlear implants or foreign metal bodies or aneurysm clips or severe claustrophobia), the inability to obtain written informed consent. For each patient, specific informed consent was collected.

### 1.5 T MR-guided radiotherapy

Before simulation (planning CT and MRI) and each fraction, patients were instructed to have their bladder half full (500 cc of water 15–20 min before the session) and an empty rectum. After consultation, all patients underwent a CT simulation scan with a slice thickness of 3-mm for dose calculation purposes, followed by a high-resolution MR scan acquired by Elekta Unity. A T2-weighted MR scan was acquired during simulation and prior to each fraction. In the case of low-risk PC, the clinical target volume (CTV) was the prostate gland only, whereas in the case of intermediate-risk PC, the entirety of the seminal vesicles (SV) was included. The planning target volume (PTV) consisted of CTV + 5 mm margins in each direction, except 3 mm posteriorly, according to previously published experiences [12]. As organs at risk, the rectum, bladder, penile bulb, urethra, and femoral heads were delineated.

The SBRT schedule consisted of five fractions of 7 Gy (total prescription dose, *D*_*p*_, equal to 35 Gy) for all patients delivered on 5 consecutive days, corresponding to normalized total doses of 2 Gy per fraction (NTD2) between 70 and 85 Gy for an α/β estimated between 3 and 1.5 Gy for PC.

The dose distribution was normalized to assure that at least 95% of the PTV received at least 95% of Dp (33.2 Gy), while less than 2% of the PTV received 107% of Dp (37.5 Gy). By considering that less than 1 cm3 of the PTV overlapping the rectum, bladder, and urethral planning-risk-volume (i.e., 3 mm isotropic expansion from the urethra) had to receive Dp, no less than 95% Dp (33.2 Gy) had to be assured to 95% of the PTV-minus-any-overlap with the rectum, bladder, or urethral PRV. However, 98% of any of such three overlapping volumes needed to receive at least 32 Gy [6].

Baseline treatment plans were generated using static field intensity modulated radiotherapy (IMRT) delivered with 16 beams.

Constraints for planning approval were the following: (1) for the rectum: V18 Gy ≤ 35%, V28 Gy ≤ 10%, V32 Gy ≤ 5%, Dmax ≤35 Gy; (2) for the bladder: Dmax ≤35 Gy; (3) for the urethral PRV Dmax ≤35 Gy. Dmax was always referred to the hottest 1 cm^3^ of the conceived organ at risk.

The two treatment plan “adaptive” strategies available for Elekta Unity are ‘adapt-to-position’ (ATP) and ‘adapt-to-shape’ (ATS). For ATP, daily delineation is neither needed nor possible, and only the (isocenter) position is modified in the pre-treatment CT. In the case of ATS, the daily MRI is re-contoured to adapt the treatment plan of the day [13].

In the PC SBRT patients group presented here, ATS was performed for all patients in every session [13]. In detail, prior to each fraction, a new T2-weighted MRI sequence (preMRi) was performed and rigidly registered to the simulation MR.

Through deformable registration, the original set of contours was projected onto the daily preMRi and hence edited, as necessary, by the physician. A full re-optimization, such as starting from fluence, was performed by the physicist and, within the second optimization phase (i.e. the segmentation phase), a second verification MRI scan was acquired to test whether the deformations of the bladder and/or rectum were negligible. If not, the patient was prepared again (by oenema and/or drinking), and only after this repositioned for treatment. If yes, the treatment was delivered with patient monitoring by cineMRi, typically acquired on two coronal and sagittal planes. At the end of the delivery, a further post-MRi scan was performed, to estimate the intra-fraction organ motion.

### Study endpoints and statistical analysis

The primary endpoint of the present analysis was a quality of life (QoL) evaluation based on PROMs. The secondary endpoint was clinician-reported toxicity measured at the last treatment session. Toxicity was assessed according to the Common Terminology Criteria for Adverse Events (CTCAE) scale, v5.0.

PROMs and QoL were investigated with the following questionnaires:

- International Prostatic Symptoms Score (IPSS)
- EORTC Quality of Life Questionnaire-Core 30 (EORTC QLQ-C30) and EORTC QLQ-PR25
- Expanded Prostate Cancer Index Composite-26 (EPIC-26)
- International Consultation on Incontinence Questionnaire- Short Form (ICIQ-SF)
- International Index of Erectile Function – 5 (IIEF-5)

IPSS scores at subsequent time points were classified into three groups as defined by the American Urological Association classification, i.e. ‘mild’ (IPSS 0–7), ‘moderate’ (IPSS 8–19), or ‘severe’ (IPSS 20–35) symptoms. The QLQ-C30 includes functional scales and single-item questions. All scales and single-item scores range from 0 to 100. A high functional scale score represents a healthy level of functioning; a high score for the global health status represents a high QoL, while a high score for a symptom scale, bowel score or urinary score represents a high level of symptomatology [14]. The European Organization for the Research and Treatment of Cancer Quality of Life Questionnaire–Prostate 25 (EORTC QLQ-PR25) is complementary to the general cancer EORTC QLQ30 questionnaire and is designed for PC patients. This questionnaire has 25 items examining urinary and bowel symptoms, sexual activity and function, and treatment-related symptoms, using a 4-point Likert response scale [15].

The Expanded Prostate Cancer Index-Composite (EPIC) is a PROM developed to monitor health-related QoL outcomes among PC patients. The 26-item version of EPIC, also known as EPIC Short Form or EPIC-26, contains five symptom domains (urinary incontinence, urinary irritative/obstructive, sexual, bowel, hormonal), scored from 0 (worst) to 100 (best), tracked over time to understand symptom burden, functional outcomes and the impact of side effect management strategies [16].

The International Index of Erectile Function – 5 (IIEF-5) questionnaire consists of only five questions and each IIEF-5 item is scored on a five-point ordinal scale where lower values represent poorer sexual function. The possible scores for the IIEF-5 range from 1 to 25, and a score above 21 was considered as normal erectile function.

The International Consultation on Incontinence Questionnaire Short Form (ICIQ-SF) is a brief PROM for evaluating the frequency, severity and impact on QoL of urinary incontinence, scored on a scale from 0 to 21.

Data analysis was performed with SPSS (version 20.0; IBM, Armonk, USA). The Wilcoxon signed rank test was applied. Significance was noted for *p*-values ≤0.05.

## Results

Between October 2019 and January 2020, 25 consecutive patients affected by low or intermediate-risk PC were treated at the Advanced Radiation Oncology Department of the IRCCS Sacro Cuore Don Calabria Hospital in Negrar, Verona, by means of 1.5 T MR-guided adaptive SBRT.

Four patients (16%) were affected by low risk PC, with the remaining 21 (84%) by favourable intermediate (11) and unfavourable intermediate (10) risk PC.

Six-months of androgen deprivation therapy was concomitantly administered in 9 (36%) unfavourable intermediate risk PC cases. One patient affected by unfavourable intermediate risk refused androgen deprivation therapy. No treatment interruption occurred.

In Table 1, the baseline patients and treatment characteristics are reported.

**Table 1:** Baseline patients’ and treatment characteristics

Age:	
- Median (years)	68
- Range (years)	54–82
PSA:	
- Median (ng/ml)	6.8
- Range (ng/ml)	1–19
Class of Risk:	
- Low	4 (16%)
- Favorable Intermediate	11 (44%)
- Unfavorable Intermediate	10 (40%)
Androgen deprivation therapy:	
- Yes	9 (36%)
- No	16 (64%)
Prostate Volume:	
- Median (cc)	36
- Range (cc)	20–61
IPSS score:	
- Median	5
- Range	0–15
Overall Treatment Time:	
- Median (minutes)	41
- Range (minutes)	20–61

For all patients analyzed herein, clinician-reported outcome measurements and PROMs were collected at the end of treatment.

### Clinician-reported outcome measurements

Early toxicity scored by clinicians according to the CTCAE scale v5.0 is shown in Table 2.

**Table 2:** Acute Toxicity Rates (CTCAE v.5)

Genitourinary (frequency, urgency, pain)	
G2: 3 (12%)	
G1: 6 (24%)	
Gastrointestinal (rectal pain)	
G2: 1 (4%)	
G1: 2 (8%)	

No grade 3 or higher acute toxicity measured by any symptom at any study time point was observed. Three patients (12%) suffered grade 2 acute genitourinary toxicity (one with urinary frequency, another with urinary tract pain, and the last with urinary retention), registered at the end of treatment. One out of these three patients had poor baseline IPSS with mild urinary tract obstruction symptoms. Only one patient (4%) experienced acute grade 2 GI at the last session of radiotherapy.

### Patient-reported outcome measurements

Patients completed the questionnaires on the first and last day of radiotherapy. All patients completed the IPSS, EORTC QLQ-C30 and QLQ-PR25, EPIC-26, ICIQ-SF and IIEF-5 questionnaires. The questionnaire had been translated into Italian according to the translation procedure of the EORTC QL Study Group.

**Fig. 1:**
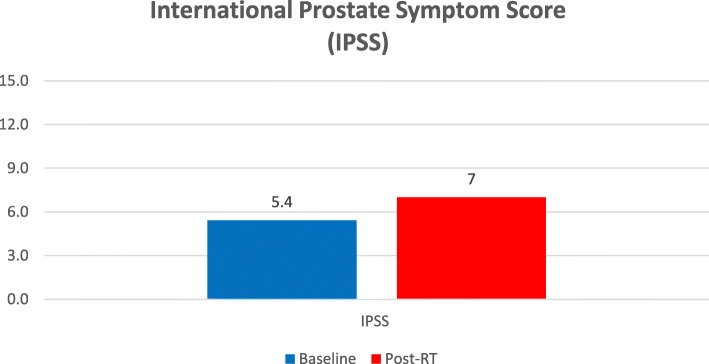
International Prostate Symptom Score (IPSS)

With regard to the IPSS scores, it is notable that 35% of patients already reported moderate symptoms (IPSS 7–10) at baseline. Median IPSS scores were 5 both at baseline and at the end of MR-guided adaptive SBRT, while mean IPSS scores were 5.4 and 7, respectively (*p* = 0.0005) (Fig. 1; Table 3).

**Table 3:** International Prostate Symptom Score (IPSS)

	Baseline (Mean ± SD)	Post-RT (Mean ± SD)	p
IPSS score	5.4 ± 3.7	7 ± 4.5	0,07

The low incidence of early grade ≥ 2 GI clinician-reported toxicity was confirmed by low bowel scores derived from the QLQ-PR25 questionnaire. The most common patient-reported GI symptom was G1 rectal pain at the end of treatment. The bowel scores derived from the QLQ-PR25 questionnaire showed no significant increase at the end of MR-guided adaptive SBRT.

**Fig. 2:**
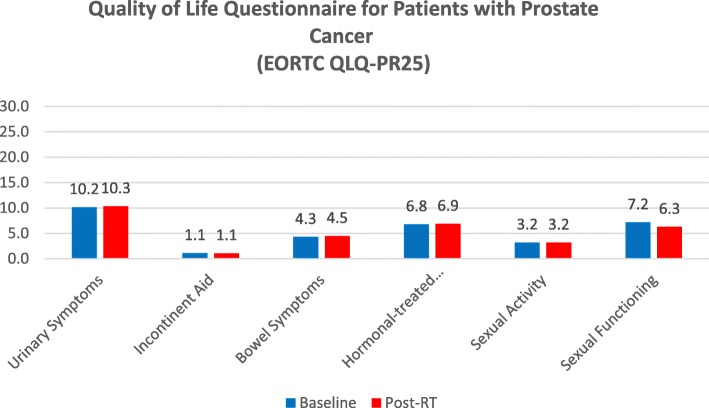
Quality of Life Questionnaire for Patients with Prostate Cancer (EORTC QLQ-PR25)

**Fig. 3:**
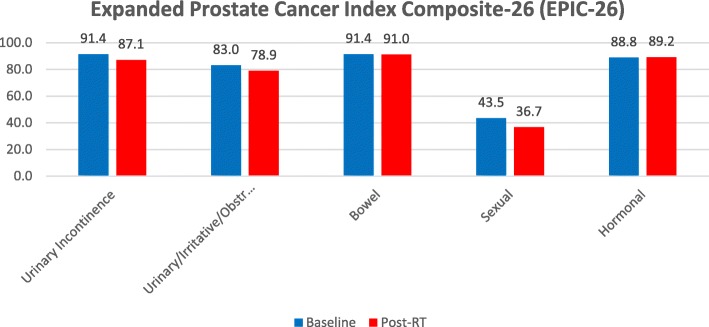
Expanded Prostate Cancer Index Composite-26 (EPIC-26)

Patient-reported urinary toxicity, assessed using the QLQ-PR25 urinary symptom scale, showed a similar pattern as the clinician-reported outcome scores with no significant increase at the end of MR-guided adaptive SBRT (Fig. 2, Table 3). Increased urinary frequency and urge symptoms were the most common early toxicity symptoms, whereas urinary incontinence was uncommon, as shown in EPIC-26 questionnaire (Fig. 3, Table 4).

**Table 4:** Quality of Life Questionnaire for Patients with Prostate Cancer (EORTC QLQ-PR25)

EORTC QLQ-PR25	Baseline (Mean ± SD)	Post-Rt (Mean ± SD)	p
Urinary Symptoms	10.2 ± 3.1	10.3 ± 3	0.21
Incontinent Aid	1.1 ± 0.5	1.1 ± 0.5	1
Bowel Symptoms	4.3 ± 0.6	4.5 ± 1.8	0.33
Hormonal-treated Related Symptoms	6.9 ± 1	6.8 ± 1.2	0.19
Sexual Activity	3.2 ± 1.7	3.2 ± 1.5	0.71
Sexual Functioning	7.2 ± 4	6.3 ± 3.4	0.76

**Fig. 4:**
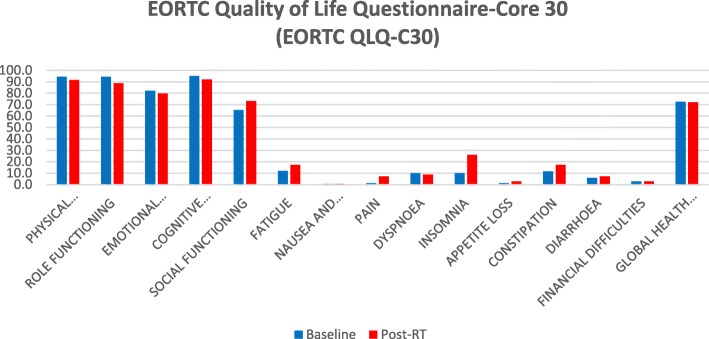
EORTC Quality of Life Questionnaire-Core 30 (EORTC QLQ-C30)

A similar trend was observed for the QoL results derived from the EORTC QLQ-C30 questionnaire (Fig. 4). None of the functional scales showed a clinically relevant difference (i.e. difference of 10 points or more) at any of the study time points with the exception of physical functioning, which decreased from 94.5% ± 10.4% at baseline to 91.6% ± 12.6% at the end of MR-guided adaptive SBRT.

Only items regarding insomnia and constipation worsened at the end of treatment compared to baseline values. Global health status values were 72.5 and 72.1% at baseline and at the end of treatment, with no statistically significant difference (Table 5).

**Table 5:** Expanded Prostate Cancer Index Composite-26 (EPIC-26)

EPIC-26	Baseline (Mean ± SD)	Post-Rt (Mean ± SD)	p
Urinary Incontinence	91.4 ± 16	87.1 ± 20.3	0.18
Urinary/ irritative/Obstructive	83 ± 15.2	78.9 ± 21.3	0.25
Bowel	91.4 ± 9.6	91 ± 14.7	0.90
Sexual	43.5 ± 25.7	36.7 ± 22.4	0.12
Hormonal	88.8 ± 12	89.2 ± 14.4	0.84

**Fig. 5:**
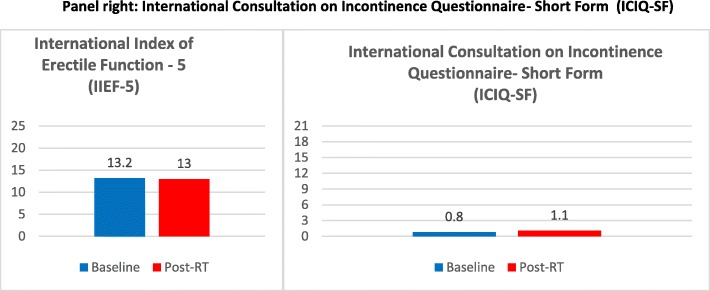
Panel left: International Index of Erectile Function – 5 (IIEF-5). Panel right: International Consultation on Incontinence Questionnaire- Short Form (ICIQ-SF)

IIEF-5 and ICIQ-SF questionnaires showed no statistically significant worsening of erectile function and urinary incontinence at the end of radiation treatment when compared to baseline values (Fig. 5, Tables 6 and 7).

**Table 6:** EORTC Quality of Life Questionnaire-Core 30 (EORTC QLQ-C30)

EORTC QLQ-C30	Baseline (Mean ± SD)	Post-RT (Mean ± SD)	p
Physical Functioning	94.5 ± 10.4	91.6 ± 12.6	0.04
Role Functioning	94.6 ± 9.9	88.8 ± 17.3	0.10
Emotional Functioning	82.2 ± 18.7	79.7 ± 16.6	0.23
Cognitive Functioning	94.9 ± 11.7	92 ± 12.2	0.16
Social Functioning	65.5 ± 40.5	73.2 ± 35.8	0.29
Fatigue	12.1 ± 15.3	17.4 ± 18.3	0.22
Nausea and Vomiting	0.7 ± 3.5	0.7 ± 3.5	0.23
Pain	1.5 ± 4.8	7.2 ± 17.3	0.12
Dyspnoea	10.1 ± 18.6	8.7 ± 20.6	0.72
Insomnia	10.1 ± 15.7	26.1 ± 26.5	0.005
Appetite Loss	1.4 ± 6.9	2.9 ± 9.6	0.33
Constipation	11.6 ± 19.1	17.4 ± 19.8	0.04
Diarrhoea	5.8 ± 12.9	7.2 ± 17.3	0.71
Financial Difficulties	2.9 ± 9.6	2.9 ± 9.6	0.23
Global Health Status	72.5 ± 13.4	72.1 ± 15.8	0.91

**Table 7:** International Index of Erectile Function – 5 (IIEF-5) and International Consultation on Incontinence Questionnaire- Short Form (ICIQ-SF)

	Baseline (Mean ± SD)	Post-RT (Mean ± SD)	p
**IIEF-5 score**	13.2 ± 7.6	13 ± 7.8	0,23
**ICIQ-SF score**	0.8 ± 2	1.1 ± 2.5	0,33

## Discussion

In the management of localized PC, the use of SBRT as primary treatment is steadily increasing. Crucial components to improve this therapeutic approach in daily clinical practice are the proper selection of patients in terms of pre-RT genitourinary functioning, adequate technological equipment, and dedicated radiation oncologists [17].

In the context of SBRT for PC, a five session schedule has been extensively investigated by several authors [17]. Few differences exist in QoL among the RT modalities, with SBRT using 35–40 Gy/5 fractions and resulting in lesser bowel QoL impact, whereas brachytherapy has a greater impact on urinary obstruction [17].

Elekta Unity is an innovative system for RT that conjugates 1.5 Tesla MRI with 7 MV FFF linac. After the installation phase in our Advanced Radiation Oncology Department, the first patient affected by localized PC was treated in October 2019. Herein, we report preliminary data in terms of clinician-reported outcome measurements and PROMs in a series of 25 localized PC patients treated by means of 1.5 T MRI-Guided adaptive SBRT using ATS workflow. Briefly, ATS (daily recontouring of structures of interest and daily adapted replanning) allows clinicians to reshape the dose based on daily changes in the shape, size and position of target volume and OARs, enabling daily accurate dose delivery with real-time visualization of the patient’s anatomy [13, 18]. Early assessment of clinician-reported outcome measurements and PROMs does not permit drawing definitive conclusions. Despite being preliminary, these data may be of great interest to the scientific community. First of all, Elekta Unity represents a new system for the oncologic community with limited worldwide diffusion, and implies new challenges for both users and patients. It is known that MRI-scanning could increase claustrophobia and anxiety in oncologic patients, affecting their quality of life [19]. Furthermore, treatment compliance and acceptance among patients could be negatively influenced by the overall fraction time. In fact, the session time is longer using adaptive MRI-guided RT compared to other devices adopted in PC RT. In our experience, the median fraction time was 56 min (range, 34–86 min). As previously reported, the ATS workflow is longer than the ATP strategy, resulting in more advanced plan adaptations that meet the clinical dose criteria [13].

To the best of our knowledge, this is the first report evaluating QoL and PROMs during 1.5 T MR-guided SBRT for prostate cancer. Similarly, Bruynzeel et al. [20] recently published an early toxicity report using a different MRI-guided SBRT (0.35 T MR, MRIdian system - ViewRay Inc.) in a large prostate cancer sample size. Regarding the PROMs, Bruynzeel et al. [20] recorded a significant worsening of the role-functioning domain.

Concerning the preliminary findings reported here, the EORTC QLQ-C30 questionnaire showed no difference from baseline to the end of treatment. More specifically, the functional scales, including emotional and role-functioning domains, were not affected by the patient’s anxiety potentially related to such complex radiation treatment procedures. Conversely, a slight impairment of the physical functioning item was noted. This domain decreased by approximately 3.1% from baseline to the end of treatment. This functional decline for prostate cancer patients is not new in active therapy. In fact, a prospective longitudinal cohort study [21] documented that, for men with newly diagnosed localised PC, physical distress was significantly more common following surgery and radiotherapy than active surveillance.

Bruynzeel et al. [20] observed low incidence of early GI and GU toxicities using 0.35 T MRI-guided SBRT for prostate cancer, both in clinician- and patient-reported outcome measurements. Specifically, the maximum cumulative grade ≥ 2 early GU and GI toxicity was 23.8 and 5.0%, respectively. In our study population, the grade ≤ 2 GU and GI toxicities were 12 and 4%, respectively. No grade 3 or higher acute toxicity was observed.

In the present analysis, the QLQ-PR25 urinary symptom scale showed a similar pattern compared to clinician-reported outcome measurement scores with no significant increase at the end of treatment. Moreover, increased urinary frequency and urgency symptoms were the most common early toxicities, whereas urinary incontinence was uncommon, as shown in the EPIC-26 questionnaire. The median IPSS scores were 5 both at baseline and at the end of MR-guided adaptive SBRT, while mean IPSS scores were 5.4 and 7, respectively (*p* = 0.0005).

Moreover, IIEF-5 and ICIQ-SF questionnaires showed no statistically significant worsening of erectile function and urinary incontinence at the end of radiation treatment. In regard to prostate SBRT, a previous study showed promising results about erectile function without significant difference from other radiotherapy techniques, evaluated by the sexual items of the EPIC-26 instrument [22].

## Conclusion

To the best of our knowledge, this is the first investigation reporting clinician-reported outcome measurements and PROMs for 1.5 T MR-guided adaptive SBRT for localized PC. The preliminary data collected here report optimal safety and tolerability, as also confirmed by PROMs questionnaires. Long-term data are warranted.

## Data Availability

The patient information may be shared under ‘IRCCS Sacro cuore – Don Calabria’ hospital IRB approval of amendment on a case by case base.

## Abbreviations

MR: Magnetic resonance
SBRT: Stereotactic body radiotherapy
PC: Prostate cancer
PROMs: Patient-reported outcomes measures
QoL: Quality of life
IPSS: International Prostatic Symptoms Score
ICIQ-SF: International Consultation on Incontinence Questionnaire- Short Form
EPIC-26: Expanded Prostate Cancer Index Composite-26
EORTC QLQ-C30: EORTC Quality of Life Questionnaire-Core 30
EORTC QLQ-PR25: EORTC Quality of Life Prostate
IIEF-5: International Index of Erectile Function – 5
CTCAE: Common Terminology Criteria for Adverse Events
IMRT-IGRT: Intensity-modulated and image-guided Radiotherapy
NNCN: National Comprehensive Cancer Network
ECOG: Eastern Cooperative Oncology Group
CT: Computed tomography
MRI: Magnetic resonance imaging
ADT: Androgen deprivation therapy
TURP: Transurethral resection of the prostate
SV: Seminal vesicles
PTV: Planning target volume
CTV: Clinical target volume
NTD2: Normalized total doses of 2 Gy per fraction
Dp: Total prescription dose
ATP: Adapt-to-position
ATS: Adapt-to-shape

## References

[1] Hamdy FC, Donovan JL, Lane JA, Mason M, Metcalfe C, Holding P (2016). 10-year outcomes after monitoring, surgery, or radiotherapy for localized prostate Cancer. N Engl J Med.

[2] Neal DE, Metcalfe C, Donovan JL, Lane JA, Davis M, Young GJ, et al. Ten-year Mortality, Disease Progression, and Treatment-related Side Effects in Men with Localised Prostate Cancer from the ProtecT Randomised Controlled Trial According to Treatment Received Eur Urol. 2019. doi: 10.1016/j.eururo.2019.10.030. 10.1016/j.eururo.2019.10.030 31771797

[3] Donovan JL, Hamdy FC, Lane JA, Mason M, Metcalfe C, Walsh E (2016). Patient-reported outcomes after monitoring, surgery, or radiotherapy for prostate Cancer. N Engl J Med.

[4] Cuccia F, Mazzola R, Arcangeli S, Mortellaro G, Figlia V, Caminiti G (2019). Moderate hypofractionated helical tomotherapy for localized prostate cancer: preliminary report of an observational prospective study. Tumori.

[5] Fersino S, Tebano U, Mazzola R, Giaj-Levra N, Ricchetti F, Di Paola G (2017). Moderate Hypofractionated Postprostatectomy volumetric modulated arc therapy with daily image guidance (VMAT-IGRT): a mono-institutional report on feasibility and acute toxicity. Clin Genitourin Cancer.

[6] Ruggieri R, Naccarato S, Stavrev P, Stavreva N, Fersino S, Giaj Levra N (2015). Volumetric-modulated arc stereotactic body radiotherapy for prostate cancer: dosimetric impact of an increased near-maximum target dose and of a rectal spacer. Br J Radiol.

[7] Dearnaley D, Syndikus I, Mossop H, Khoo V, Birtle A, Bloomfield D (2016). Conventional versus hypofractionated high-dose intensity-modulated radiotherapy for prostate cancer: 5-year outcomes of the randomised, non-inferiority, phase 3 CHHiP trial. Lancet Oncol.

[8] de Vries KC, Wortel RC, Oomen-de Hoop E, Heemsbergen WD, Pos FJ, Incrocci L (2020). Hyprofractionated versus conventionally fractionated radiation therapy for patients with intermediate- or high-risk, localized, prostate Cancer: 7-year outcomes from the randomized, multicenter, open-label, phase 3 HYPRO trial. Int J Radiat Oncol Biol Phys.

[9] National Comprehensive Cancer Network (NCCN) (2014). Clinical practice guidelines in oncology.

[10] Brand DH, Tree AC, Ostler P, van der Voet H, Loblaw A, Chu W (2019). Intensity-modulated fractionated radiotherapy versus stereotactic body radiotherapy for prostate cancer (PACE-B): acute toxicity findings from an international, randomised, open-label, phase 3, non-inferiority trial. Lancet Oncol.

[11] Nicosia L, Mazzola R, Rigo M, Figlia V, Giaj-Levra N, Napoli G (2019). Moderate versus extreme hypofractionated radiotherapy: a toxicity comparative analysis in low- and favorable intermediate-risk prostate cancer patients. J Cancer Res Clin Oncol.

[12] Alongi F, Mazzola R, Fiorentino A, Corradini S, Aiello D, Figlia V (2019). Phase II study of accelerated Linac-based SBRT in five consecutive fractions for localized prostate cancer. Strahlenther Onkol.

[13] Winkel D, Bol GH, Kroon PS, van Asselen B, Hackett SS, Werensteijn-Honingh AM (2019). Adaptive radiotherapy: The Elekta Unity MR-linac concept. Clin Transl Radiat Oncol.

[14] Fayers PM, Aaronson NK, Bjordal K, on behalf of the EORTC Quality of Life Group (2001). EORTC QLQ-C30 Scoring Manual.

[15] van Andel G, Bottomley A, Fosså SD (2008). An international field study of the EORTC QLQ-PR25: a questionnaire for assessing the health-related quality of life of patients with prostate cancer. Eur J Cancer.

[16] Szymanski KM, Wei JT, Dunn RL, Sanda MG (2010). Development and validation of an abbreviated version of the expanded prostate cancer index composite instrument for measuring health-related quality of life among prostate cancer survivors. Urology..

[17] De Bari B, Arcangeli S, Ciardo D, Mazzola R, Alongi F, Russi EG (2016). Extreme hypofractionation for early prostate cancer: biology meets technology. Cancer Treat Rev.

[18] Corradini S, Alongi F, Andratschke N, Belka C, Boldrini L, Cellini F (2019). MR-guidance in clinical reality: current treatment challenges and future perspectives. Radiat Oncol.

[19] Dewey M, Schink T, Dewey CF (2007). Claustrophobia during magnetic resonance imaging: cohort study in over 55,000 patients. J Magn Reson Imaging.

[20] Ame B, Tetar SU, Oei SS, Senan S, Haasbeek CJA, Spoelstra FOB (2019). A prospective single-arm phase 2 study of stereotactic magnetic resonance guided adaptive radiation therapy for prostate Cancer: early toxicity results. Int J Radiat Oncol Biol Phys.

[21] van Stam MA, Aaronson NK, Bosch JLHR, Kieffer JM, van der Voort van Zyp JRN, Tillier CN (2020). Patient-reported Outcomes Following Treatment of Localised Prostate Cancer and Their Association with Regret About Treatment Choices. Eur Urol Oncol.

[22] Dess RT, Hartman HE, Aghdam N, Jackson WC, Soni PD, Abugharib AE (2018). Erectile function after stereotactic body radiotherapy for localized prostate cancer. BJU Int.

